# The progressive control of foot-and-mouth disease (FMD) in the Republic of Kazakhstan: Successes and challenges

**DOI:** 10.3389/fvets.2023.1036121

**Published:** 2023-04-17

**Authors:** Akhmetzhan A. Sultanov, Samat Tyulegenov, Gulzhan N. Yessembekova, Maksat A. Berdikulov, Yersyn Mukhanbetkaliyev, Amina Akhmetzhanova, Andres M. Perez, Sarsenbay K. Abdrakhmanov

**Affiliations:** ^1^Kazakh Research Veterinary Institute, Almaty, Kazakhstan; ^2^S. Seifullin Kazakh Agrotechnical University, Astana, Kazakhstan; ^3^Republican State Enterprise “National Veterinary Reference Center,” Committee of Veterinary Control and Supervision of the Ministry of Agriculture of the Republic of Kazakhstan, Astana, Kazakhstan; ^4^Kazakh National Agrarian Research University, Almaty, Kazakhstan; ^5^Department of Veterinary Population Medicine, University of Minnesota Twin Cities, St. Paul, MN, United States

**Keywords:** Kazakhstan, foot-and-mouth disease, vaccination, control, epidemiology

## Abstract

Foot-and-mouth disease (FMD) has historically caused far-reaching economic losses to many regions worldwide. FMD control has been problematic, and the disease is still prevalent in many West and Central Asia countries. Here, we review the progress made by Kazakhstan in achieving freedom from FMD and discuss some of the challenges associated with maintaining the FMD-free status, as evidenced by the occurrence of an outbreak in 2022. A combination of zoning, movement control, vaccination, and surveillance strategies led to eliminating the disease in the country. However, the circulation of the FMD virus in the region still imposes a risk for Kazakhstan, and coordinated strategies are ultimately needed to support disease elimination. The results presented here may help design effective pathways to progressively eliminate the disease in West and Central Asia while promoting the design and implementation of regional actions to support FMD control.

## Introduction

Foot-and-mouth disease (FMD) is an infectious disease of cloven-hoofed animals caused by the infection with a picornavirus generically referred to as the FMD virus (FMDv). FMD causes far-reaching losses to affected countries (1, 2). Although some regions have made substantial progress toward controlling the disease, most countries have not reached FMD-free status, as described by the World Organization for Animal Health (WOAH).

Much progress has been made since the inception of the West Eurasia Roadmap for FMD Control in 2008, and the 14 countries included in the regional effort have made some level of progress toward the progressive control of the disease (3). Kazakhstan is the only country in the region that has achieved FMD-free status, as recognized by WOAH in 2017. However, FMD is believed to still be present in many countries of the region, which represents a threat to Kazakhstan. For example, Mongolia and China have consistently reported serotype A and O FMD outbreaks to WOAH (https://wahis.woah.org/) almost annually over the last 10 years. In Russia, outbreaks of FMD caused by the O/ME-SA/Ind-2001 virus were first registered in Zabaykalsky Krai, Russia, in 2016 and 2019 and the Orenburg region, close to the border with Kazakhstan in 2021. In 2022, an FMD outbreak was reported in Kazakhstan, resulting in the provisional suspension of the disease-free status of the affected zone. A description of the outbreaks reported in 2022 is available elsewhere (4). Briefly, the outbreak was initially suspected through passive surveillance. In response to the emergency, Kazakhstan initiated a national vaccination campaign, which resulted in the suspension of the FMD-free status without vaccination in the zones that previously had such status. Although the cost that FMD causes to Kazakhstan is unknown, noteworthy, the federal government is responsible for the cost of control and prevention activities and for compensating producers at live market value. The value of 1 kg of beef and 1 kg of live animal is, approximately, USD 5.4 and USD 2.6 per kg, respectively. Thus, culling of, say, 10 cattle of, on average, 350 kg each, will cost USD 9,100 to the federal government in terms of compensation and will represent a loss of approximately USD 9,800 to the affected producer due to the differential price. Those estimates do not include other losses, such as those associated with the genetic and productive value of lost animals, cost of disease control activities (e.g., vaccination and movement restrictions), and loss of export markets.

The remarkable success of Kazakhstan in achieving the FMD-free status in the Western Asia region, followed by the subsequent loss of the status in association with a new FMD incursion, is of interest because it represents an example of the potential opportunities and risks associated with the control of FMD in the region.

The objective of the paper here was to review the evolution of the FMD control program in Kazakhstan and to offer a discussion of the emerging challenges toward eliminating the disease in the country. The results and discussion here will be helpful for the design and implementation of effective FMD control programs in the region.

## Demographic features of Kazakhstan

**Figure 1:**
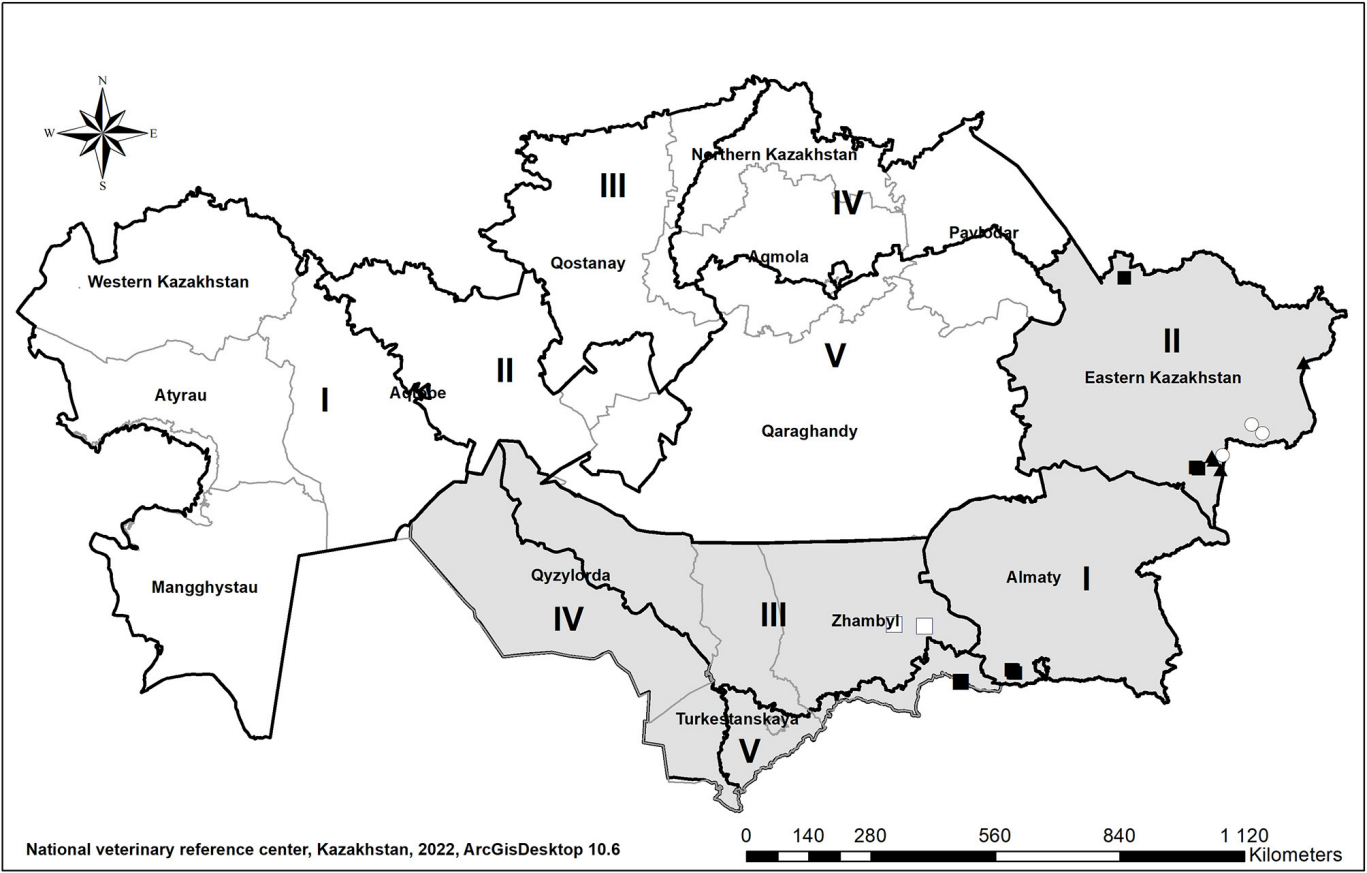
The fourteen administrative units of Kazakhstan (thin borders) grouped into ten zones (thick borders designated in Roman, I–V, numerals) according to their foot-and-mouth disease (FMD) status (gray: with vaccination; white: without vaccination), as approved by the World Organization for Animal Health (WOAH) in 2019. FMD outbreaks reported in 2011 (triangles), 2012 (squares), and 2013 (circles) caused by serotype O (black) and serotype A (white) FMD viruses are also indicated.

Kazakhstan is the largest land-locked country in the world, resulting in more than 14,000 km of borders with five neighboring countries. Administratively, the country is divided into 14 regions (Figure 1). The agricultural industry is vital for Kazakhstan, with almost 50% of the country's population living in rural areas and approximately one-third of the population directly or indirectly associated with the agricultural sector. Also, ~75% of all agricultural land is used for grazing, mostly, ruminants susceptible to FMD infection (5).

Environmental conditions and animal production features vary regionally in Kazakhstan.

In Western Kazakhstan, an area rich in large meadows and pastures, animal production tends to be more extensive and most often includes sheep, and horses. Because of the relatively low animal density, extensive conditions, and relative isolation, FMD outbreaks are relatively rare and self-limiting in this region. In Northern Kazakhstan, production is typically relatively intensive, and much of the swine and dairy production in the country is located here. The livestock industry is expanding in this region, primarily because of the interest in exporting dairy products. There are plans to build 52 dairy farms here in the following years, at a rate of 10–15 farms per year, and the federal government has already allocated 3 billion tenges (~USD 6.5 million) for the construction of some initial dairy farms. Because of the relatively extensive production in Western Kazakhstan and the intentions to develop the industry to target exports from Northern Kazakhstan, the objective of reaching FMD-free status without vaccination was considered for those regions.

In turn, prevailing high-temperature conditions in the foothills of Southern Kazakhstan result in the production of livestock adapted to those conditions, most importantly, small ruminants. In Eastern Kazakhstan, non-irrigated agriculture is relatively standard and beef and dairy cattle farms are rapidly growing mainly to provide the Kazakh internal market with dairy products and beef. Approximately seventy dairy farms and a hundred feedlots operate in Eastern Kazakhstan. Because of those environmental and demographic conditions, Eastern and Southern Kazakhstan are considered at higher risk for FMD than other parts of the country.

Maps depicting the density of cattle, small ruminants, and pigs in Kazakhstan, along with population data, have been provided in Supplementary material.

## Epidemiological pattern of FMD in Kazakhstan prior to the achievement of the FMD-free status (1955–2013)

A detailed description of the epidemiological dynamics of FMD infection in Kazakhstan is available elsewhere (6). Briefly, 5,260 serotype O and A FMDv outbreaks were recorded in Kazakhstan between 1955 and 2013. Most (>75%) outbreaks affected cattle. FMD outbreaks were spatiotemporally clustered before 1970, with two seasonal peaks (in spring and fall). Between 1984 and 2013, outbreaks occurred only sporadically and in spring, with clusters associated only with the incursion of specific variants of serotype A FMDv.

The risk for disease incursions into the Southern part of the country became evident when a series of outbreaks, caused by various serotypes and strains, including A SEA 97, A Iran 05, O PanAsia, and O / PanAsia 2, were reported in these zones between 2011 and 2013 (Figure 1). During that period, outbreaks were controlled through a mass vaccination program that resulted in the application of 16.5 million and 6.3 million FMD vaccine doses in cattle and small ruminants, respectively. The vaccine included FMDv strains Asia-1 Shamir, Manisa type 01 and Iraq type A22.

## Implementation of the pathway for an FMD control program (2013–2022)

A strategic plan for FMD control was designed according to WOAH recommendations and considering the social, demographic, and epidemiological features of the disease and setting and implemented following order No. 7-1/587 of the Minister of Agriculture of the Republic of Kazakhstan, dated June 29, 2015 (7). The plan was based on a combination of zoning, preventive vaccination followed by the serological evaluation of population immunity, and control of movements between zones. Key pillars of the plan also included (a) all costs, including vaccination, laboratory testing, elimination of positive animals, and compensation, were publicly funded; (b) engagement of the entire network of veterinary diagnostic laboratories in the country, the National Reference Center for Veterinary Medicine and the Kazakh Scientific Research Veterinary Institute; (c) implementation of animal identification and movement tracking system; and (d) agreement with neighboring countries to strengthen surveillance and inspection activities at the borders, including mobile checkpoints, the creation of bi-national and multi-national committees to monitor the epidemiological conditions and share information on outbreaks, and joint implementation of surveillance activities. The state compensated farmers for destroying sick and in-contact animals at market value for 1,118,076,416 tenges or 7.6 million U.S. dollars in 2011–2013 only. The strategy was successful in helping Kazakhstan evolve from stage 1 of the Progressive Control Pathway for FMD, PCP-FMD (8) in 2013 to the recognition of the 14 administrative regions of the country as FMD-free in 2017-−9 and 5 with and without vaccination, respectively (3).

The 14 administrative units of Kazakhstan were grouped into 10 zones according to their FMD status, half corresponding to zones with and without vaccination (Figure 1). The five FMD-free zones without vaccination included West Kazakhstan, Atyrau, Mangystau regions and the southwestern part of the Aktobe region (zone I), the north-eastern part of the Aktobe region, the southern part of Kostanay region and the western part of Karaganda region (zone II), the northern and central part of Kostanay region, the western part of North Kazakhstan and Akmola regions (zone III), the central and eastern part of North Kazakhstan region, the northern part of Akmola and Pavlodar regions (zone IV), and the central and eastern part of Karaganda region, the southern part of Akmola and Pavlodar regions (zone V). The five FMD-free zones with vaccination included Almaty (zone I), East Kazakhstan (zone II), part of the Kyzylorda region, the northern part of South Kazakhstan region, the northern and central parts of Zhambyl region (zone III), the southern part of Kyzylorda region and the southwestern part of South Kazakhstan region (zone IV), and the southeastern part of South Kazakhstan region and the southern part of Zhambyl region (zone V).

The decision to maintain the vaccination program in Eastern and Southern Kazakhstan was due to the combination of observed demographic, environmental, and epidemiological conditions, resulting in high FMD risk levels compared to other regions. Specifically, FMD outbreaks have been relatively uncommon in the Northern and Western districts of the country (Table 1). In contrast, results of the epidemiological investigations of outbreaks reported in Southern and Eastern Kazakhstan suggested that they were associated with incursions from neighboring countries. Transmission between countries in the region, including the neighboring countries of China and Russia, and also Mongolia, may be explained by the strong social and economic relations among populations. In Mongolia, FMDV O/ME-SA/Ind-2001 was first identified in March 2015 in the westernmost region of Bayan-Olgii. In 2021, multiple FMD outbreaks caused by the O/ME-SA/Ind-2001 genetic lineage virus were registered, covering 19 of 21 regions of Mongolia, and causing outbreaks among wild Mongolian gazelles (*Procapra gutturosa*) (https://wahis.woah.org/#/in-review/3800?reportId=158431&fromPage=event-dashboard-url). Many Kazakhs live in the Bayan-Ulgiy region of Mongolia and in Russia, maintaining close ties with relatives in Kazakhstan. This situation may result in the introduction of FMD and other diseases through vehicles and contaminated food and supplies. For those reasons, it was perceived that Eastern and Southern Kazakhstan were at the highest risk for FMD introduction.

**Table 1:** Epidemiological features of the last foot-and-mouth disease (FMD) outbreaks reported in Kazakhstan in each control zone and before 2022.

**FMD status**	**Zone**	**Last FMD incursion prior to 2022**			
		**Districts affected (year)**	**Number of outbreaks**	**Serotype**	**Number of animals (cattle and small ruminants) culled**
Free without vaccination	I	Tinali and Lbischensk Akzhaik (2011)	2	O	4,299
	II	Kobda (1969)	1	O	Not available
	III	FMD has never been recorded, at least since 1955			
	IV	Yereymentau (2010)	1	O	2,025
	V	Zhezkazgan (2007)	1	O	60
Free with vaccination	I	Districts bordering China and Kyrgyzstan in Almaty (2012)	4	O	1,698
	II	Various districts (2011–2013)	14	O and A	18,869
	III	Districts bordering Kyrgyzstan in Zhambyl (2012)	3	A	270
	IV	FMD has never been recorded, at least since 1955			
	V	Kordai (2012)	1	O	270

Additionally, as described above, Southern and Eastern Kazakhstan are densely populated with small ruminants, and their products supply the internal market; thus, there was an intention to actively mitigate that risk through preventive vaccination. In turn, production in the Western region of the country is extensive, with little opportunity for disease transmission, and there is an intention and motivation to create appropriate conditions for exporting in the Northern region. For those reasons, FMD vaccination has been maintained in Southern and Eastern regions to serve as a buffer for the rest of the country, whereas it has been banned in Northern and Western parts. Consequently, Kazakhstan also becomes a major buffer between Eastern and Central Asia, and Russia and Eastern Europe, preventing the spread of FMD into free regions.

In coordination with WOAH's sub-regional office, which was established in Astana, Kazakhstan, in 2013, and to support the recognition of the FMD-free status, Kazakhstan requested WOAH to conduct Performance of Veterinary Services (PVS) evaluation missions in 2016 and 2018, which helped to identify strengths and areas for improvement with the final objective of strengthening the ability of the veterinary services to implement the measures required to control and prevent the introduction of the disease. Areas identified as key for the success of the program and in light of the results of the PVS were the structure of the veterinary service, including control, supervisory bodies, and executive bodies, a developed network of accredited veterinary laboratories, and the allocation of a national budget to support preventive measures. Additionally, simulation exercises were conducted in Karaganda and West Kazakhstan regions with the support of international experts and representatives from WOAH subregional office to improve the effectiveness of early detection and control activities.

## Vaccination, active surveillance, and evaluation of immunity to support the FMD-free status

The FMD vaccination campaign is supervised by the Minister of Agriculture of the Republic of Kazakhstan through order No. 7-1/587, which regulates the provisions of subparagraph 6, Article 8 of the Law of the Republic of Kazakhstan No. 339 (“On Veterinary Medicine”) approved in July 2002. These regulations align with the list of selected animal diseases prevention, diagnosis, and control, which is conducted at the expense of national funds, approved by order of the Minister of Agriculture of the Republic of Kazakhstan No. 7-1/559, dated October 30, 2014. Because of the extensive borders of Kazakhstan with countries in which the disease is present or suspected and in response to the 2011–2013 epidemic, since 2014, a mass vaccination campaign for cattle, small ruminants, and pigs has been implemented using a trivalent (A, O, and Asia-1) purified vaccine produced by the FFE “Shchelkovo Biocombine” and FGBI “ARRIAH” (Russia) with the activity of at least 6PD50 for each valency in a dose. The FMD mass vaccination campaign covers all susceptible animals in the five FMD-free zones where vaccination is practiced, representing approximately 14.7, 67.5, and 1.6% of the total number of cattle, small ruminants, and pigs of the country, respectively. Adult (>18-month-old) animals are vaccinated twice per year, in spring (April–May) and fall (September-October), whereas young (3–18 month-old) animals are vaccinated every 3 months.

In order to quantify the efficacy and coverage of the vaccination campaign, as well as the quality of the vaccine, post-vaccination monitoring was conducted annually among susceptible animals (cattle, small ruminants, and pigs) using an ELISA test and including at least 1% of the estimated population of vaccinated animals.

The level of immunity raised against all FMD serotypes and across the five FMD with vaccination regions was >80% after 2013 and >90% after 2015, which was considered sufficient to prevent FMD spread. In contrast, the immunity levels were substantially low (and for some serotypes and regions, <80%) prior to 2014, which may explain, at least in part, the occurrence of 15 FMD outbreaks in the region from 2011 through 2013. The number of tested animals (cattle, small ruminants, pigs) ranged from 135,900 in 2016 to 332,400 in 2017 (Supplementary material).

Additionally, screening for non-structural protein (NSP) antibodies was conducted using an ELISA test for young (3–12 month-old) livestock. The number of tested animals was calculated using a two-stage random sampling design and following guidelines provided in Chapter 1.4.4. of WOAH Terrestrial Code (9). The selection of units was implemented by the consecutive and random identification of villages, herds, and animals to sample. The total number of blood serum samples, which was stratified per zone both in the regions with and without vaccination, was 109,192 in 2016 (69,352 in the zones with vaccination, of which 36 were NSP positive, and 39,840 in the zones without vaccination); 54,138 in 2017 (14,658 in the zones with vaccination, of which 18 were NSP-positive and 39,480 in the zones without vaccination); 48,928 in 2018 (11,920 in the zones with vaccination, of which 23 were NSP-positive, and 37,008 in the zones without vaccination); 44,450 in 2019 (19,929 in the zones with vaccination, of which 13 were NSP positive and 24,519 in the zones without vaccination). Probang samples were collected from NSP-positive animals and tested by PCR; all animals tested negative. No NSP-positive result was found in animals sampled in the zones without vaccination.

## Discussion: Challenges and opportunities

FMD control is critically important to support the development of countries. FMD impact on countries' economies is associated with a combination of (1) direct losses due to reduced production and changes in herd structure; and (2) indirect losses caused by costs of FMD control, poor access to markets and limited use of improved production technologies. Although we failed to identify accurate and current estimates of the impact of FMD in Kazakhstan, it was estimated in 2013 that the annual median FMD impact on Asian countries (excluding China and India) is approximately USD 1.3 billion, considering production losses and vaccination costs only (2). Although the nature of the relationships between reaching FMD-free status and trade is difficult to measure, it is worth to note that the volume of exported meat and meat products in 2017, when Kazakhstan first reached the FMD-free status in 14 administrative regions, was 4,154 tons, whereas by 2022, this value was 27,402 tons, representing a 6.6 fold increase (Kazakhstan government, unpublished data).

A key component of Kazakhstan's success in controlling FMD may have been the allocation of sufficient financial and human resources to support the plan. As described elsewhere, the financial resources allocated to Asian veterinary services have been inadequate, impairing the effectiveness of FMD control and elimination in the region (10, 11). Despite the remarkable success of Kazakhstan in establishing an effective FMD control program, becoming the first country in the region to be recognized as disease-free by WOAH, there is still a high risk for disease incursions, as evidenced by the occurrence of an FMD outbreak in January 2022, in the FMD-free zone (V) where vaccination is not practiced. The epidemic resulted in the suspension of Kazakhstan's FMD-free status. Further investigation of the incursion revealed that the outbreak was caused by a strain that had been previously identified in neighboring countries, demonstrating the need for regional policy and actions intended to secure the free status of neighboring countries and to prevent the transboundary spread of the disease (4). Such need is not unique to the Central Asia region. Strengthening veterinary services, political will and cooperation, technical expertise, and human resources to achieve compliance with controls are also key components of FMD control, as identified in South East Asia and South America (12, 13).

The country's investment to support the control has been key to engage producers. Given that vaccine and vaccination costs were covered by the country and delivered entirely free of charge to producers, and that outreach activities were organized to engage farmers, there was strong support from producers to the FMD control campaign. Implementation of an accurate traceability system supported by the country to monitor and control animal movements was also critically important. Another relevant incentive for the private sector was the investment in technology for producers to enhance livestock productivity and reach international markets. Those actions led to a bilateral agreement with China, which required FMD-free status for trade, but that considered the regionalization plan proposed and implemented by Kazakhstan (Kazakhstan government, unpublished data).

Results here demonstrate, the opportunity to succeed in the implementation of a PCP-FMD in the West Asia region, as suggested by Kazakhstan's achievements in obtaining support from producers for the implementation of the control plan, and recognition as a disease-free country, and the need for ongoing active monitoring of the disease status in-country, and also, advancing in strategies coordinated among countries in West and Central Asia toward the ultimate goal of eliminating FMD in the region.

## Data availability statement

The original contributions presented in the study are included in the article/Supplementary material, further inquiries can be directed to the corresponding authors.

## Author contributions

AS: secured the data, planned much of the study, conceived the study, supervised activities, and wrote some of the paper. AP: collaborated with the design of the paper and wrote some of the paper. ST, GY, MB, YM, and AA: collected and organized data and wrote some of the paper. SA: conceived the study, supervised activities, and wrote some of the paper. All authors contributed to the article and approved the submitted version.
