# Supplementary material for: The progressive control of foot-and-mouth disease (FMD) in the Republic of Kazakhstan: Successes and challenges
Source: Front Vet Sci. 2023 Apr 17;10:1036121. doi: 10.3389/fvets.2023.1036121 (PMC10149985; doi:10.3389/fvets.2023.1036121)
Supplement: Supplementary file 1 [file Data_Sheet_1.docx]

**Supplementary file**

1. **Livestock demographics in Kazakhstan**

Number of agricultural presmises by regions of Kazakhstan as of April 2021 (excluding forestry and fisheries)

| **Region** | **Number of agricultural premises** |
| --- | --- |
| Turkestan region | 3695 |
| Alma-Ata's region | 2122 |
| Akmola region | 1999 |
| North-Kazakhstan region | 1346 |
| The East Kazakhstan region | 1241 |
| Kostanay region | 928 |
| Jambyl Region | 908 |
| Almaty city | 814 |
| Karaganda region | 757 |
| Shymkent | 672 |
| West-Kazakhstan region | 629 |
| Aktobe region | 592 |
| Pavlodar region | 574 |
| Astana | 554 |
| Kyzylorda Region | 529 |
| Mangistau region | 167 |
| Atyrau region | 142 |

*Source:* https://marketingcenter.kz/20/rynok-selskoe-khoziaistvo-kazakhstan.html#skhpogolovie

| **Animal species** | **2017** | **2018** | **2019** | **2020** | **2021** | **2022** |
| --- | --- | --- | --- | --- | --- | --- |
| Cattle | 6 413,2 | 6 764,2 | 7 150,9 | 7 436 | 7 850 | 9 433,3 |
| Pigs | 834,2 | 815,1 | 798,7 | 813,2 | 816,7 | 841,2 |
| Sheep | 15 884,8 | 16 049,8 | 16 416,1 | 16 912,9 | 17 749,5 | 22 240,8 |
| Goats | 2 299,4 | 2 279,1 | 2 282,9 | 2 242,7 | 2 307,9 | 2 720,1 |
| Horses | 2 259,2 | 2 415,6 | 2 646,5 | 2 852,2 | 3 139,8 | 3 935,4 |
| Poultry | 36 910 | 39 913,4 | 44 337,9 | 45 041,4 | 43 334,9 | 49 751,8 |
| Camels | 180,0 | 193,1 | 207,5 | 216,3 | 227,7 | 268,7 |

*Source:* Statistical bulletin "Major indicators of livestock development" (Agency of statistics of the Republic of Kazakhstan http://stat.gov.kz)

**Population density of cattle**


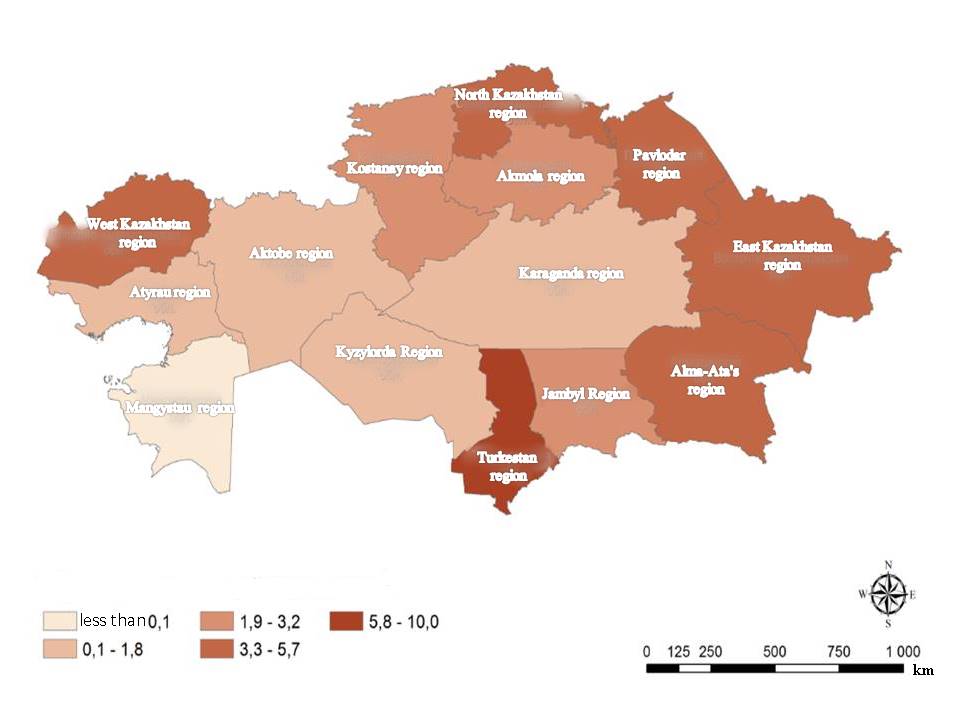


**Population density of small ruminants**


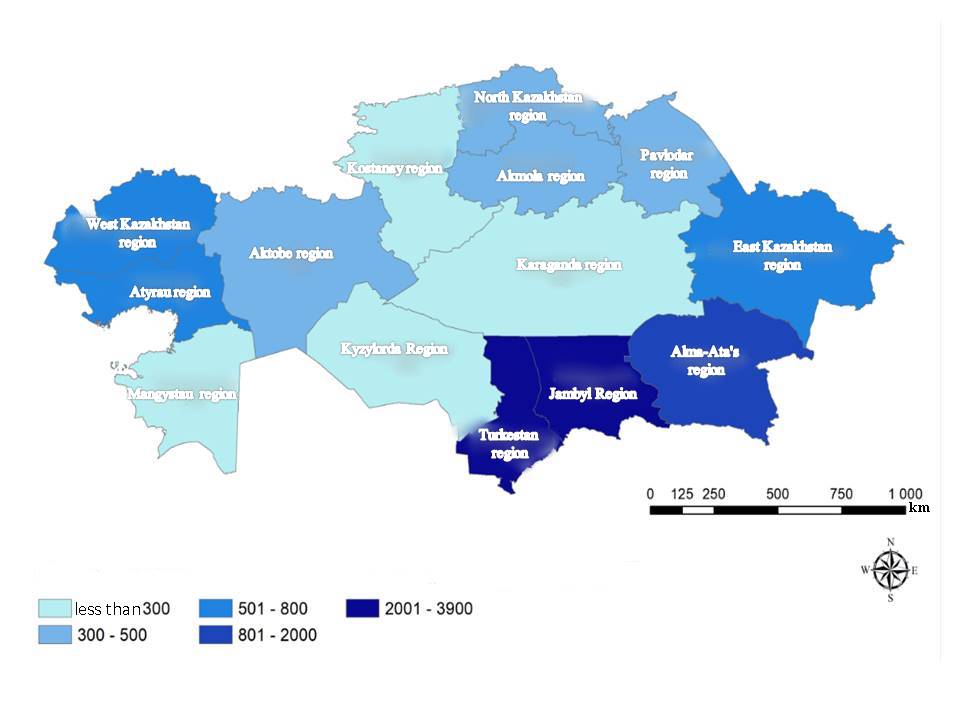


**Population density of pigs**


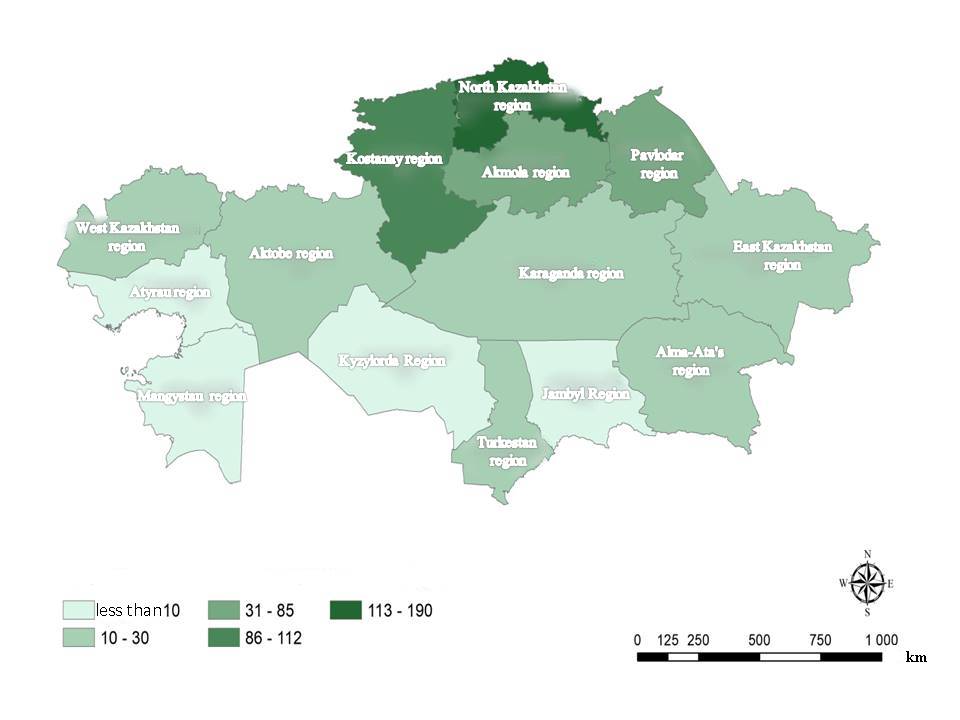


**2) Results of foot-and-mouth disease (FMD) post-vaccination monitoring in Kazakhstan** **(2016-2017)**

| **The Year** | **Type of animal** | **Antibody type** | | | | | | | | |
| --- | --- | --- | --- | --- | --- | --- | --- | --- | --- | --- |
|  |  | **Number of animals examined** | **Type A** | | | **Type O** | | **Type Asia-1** | | **Average %** |
|  |  |  | **number of immune** | | **% immunity** | **number of immune** | **% immunity** | **number of immune** | **% immunity** |  |
| **2016** | Cattle | 32 912 | 32 854 | 99,82 | | 32 880 | 99,90 | 32 858 | 99,84 | 99,85 |
|  | Small cattle | 102561 | 102452 | 99,89 | | 102364 | 99,81 | 102362 | 99,81 | 99,84 |
|  | pigs | 427 | 426 | 99,77 | | 426 | 99,77 | 427 | 100 | 99,84 |
| **Total** | | **135900** | **135732** | **99,88** | | **135670** | **99,83** | **135647** | **99,81** | **99,84** |
| **2017** | Cattle | 79 342 | 73 718 | 71 724 | | 72 459 | 92,91 | 90,40 | 91,32 | 91,55 |
|  | Small cattle | 252786 | 233782 | 232188 | | 235076 | 92,48 | 91,85 | 92,99 | 92,44 |
|  | pigs | 291 | 265 | 256 | | 265 | 91,07 | 87,97 | 91,07 | 90,03 |
| **Total** | | **332419** | **307765** | **304168** | | **307800** | **92,58** | **91,50** | **92,59** | **92,23** |
